# Digital Twins for Personalized Medicine Require Epidemiological Data and Mathematical Modeling: Viewpoint

**DOI:** 10.2196/72411

**Published:** 2025-08-05

**Authors:** Alexandre Vallée

**Affiliations:** 1 Department of Epidemiology and Public Health Foch Hospital Suresnes France

**Keywords:** digital twin, personalized medicine, epidemiology, clinical practice, machine learning, mathematical modeling, health care simulation, omics data integration, predictive analytics, graph neural networks, risk prediction, big data, health care, public health surveillance, artificial intelligence, AI

## Abstract

Digital twin (DT) technology is revolutionizing clinical practice by integrating diverse epidemiological data sources to create dynamic, patient-specific simulations. By leveraging data from genomics, proteomics, imaging, sociodemographics, and real-world behaviors, DTs provide a computational framework to model disease progression, optimize treatments, and personalize health care interventions. Through artificial intelligence (AI) and mathematical modeling, DTs facilitate predictive analytics for disease risk assessment, early diagnosis, and treatment response forecasting. This viewpoint explores the mathematical foundations of DTs, including differential equations for health trajectory modeling, Bayesian networks for multiomics integration, Markov models for disease progression, and reinforcement learning for treatment optimization. In addition, machine learning techniques such as recurrent neural networks and transformers enhance the predictive power of DTs by analyzing time-series clinical data and predicting future health events. The potential applications of DTs extend beyond individual patient care to public health surveillance, hospital resource management, and epidemiological modeling. However, several challenges persist, including data privacy concerns, computational infrastructure requirements, validation of predictive models, and regulatory compliance. Addressing these limitations requires interdisciplinary collaboration among health care providers, data scientists, and policy makers. With advancements in AI, wearable technology, and multiomics data integration, DTs are poised to reshape precision medicine. Future research should focus on refining computational efficiency, standardizing data interoperability, and ensuring ethical AI-driven decision-making. The continued evolution of DTs offers a transformative approach to proactive and personalized health care, reducing disease burden and enhancing patient outcomes.

## Background

Digital twins (DTs) represent one of the most promising yet underrealized innovations in modern medicine. More than just virtual models, DTs offer dynamic simulations of patient health that integrate real-time data, artificial intelligence (AI), and computational modeling [1,2]. While their origins lie in engineering, their potential for transforming clinical practice is now undeniable [3,4]. Most existing approaches to DTs in medicine emphasize computational performance, data fusion, or sensor-based reactivity. However, these developments often lack grounding in population-level health data or validated predictive frameworks that ensure clinical generalizability.

In clinical practice, the implementation of DTs relies on integrating diverse and complex datasets [5]. These datasets include structured data such as laboratory test results, imaging scans, genomic sequencing, and electronic health records, as well as unstructured data from wearable devices, environmental exposures, and behavioral patterns. The fusion of these data streams enables the creation of a comprehensive, patient-specific model that can simulate health outcomes and propose optimized treatment plans [5]. The potential applications of DTs extend beyond individual patient care to broader public health initiatives [6]. By aggregating anonymized DT data from populations, health care providers and researchers can identify emerging disease patterns, optimize resource allocation, and develop targeted intervention strategies [7,8].

The predictive power of DTs also extends to epidemiological modeling, allowing for improved disease surveillance and response planning [9]. However, it is precisely through the integration of epidemiological modeling, longitudinal datasets, probabilistic inference, and risk stratification that DTs can evolve from theoretical constructs to trusted clinical tools. This perspective remains underrepresented in the current literature despite its critical importance to ensuring that DTs serve not just individuals but also public health systems at large. Thus, to advance DTs in clinical medicine, we should move beyond technological enthusiasm and engage critically with these structural limitations. This viewpoint outlines a mathematical and epidemiological framework for DTs, critiques current barriers to clinical implementation, and advocates for a translational model grounded in real-world health data.

## DT Concept

Since the early 2000s, the advent of new technologies such as sensors and connected objects has changed the way in which data are exchanged between different sources, generating an increasing production of metadata. Today, scientific advances in metadata analysis and the integration of AI allow for the storage and processing of these new data, paving the way for the potentially significant dissemination of one of the most intriguing technological advancements: DTs.

The concept of DTs is based on the creation of virtual alter egos of objects, living organisms, spaces, or processes. They originate from mirrored systems or simulated environments created by the National Aeronautics and Space Administration in the 1970s to monitor inaccessible physical spaces (such as spacecraft on missions) [10]. The most famous early application of DTs occurred during the Apollo 13 mission, when engineers had to guide astronauts in constructing an improvised air purifier using materials available in the spacecraft to bring the Apollo 13 crew back to Earth. This example is considered the precursor of DTs, enabling the connection between physical and virtual spaces [11,12].

It was only in 2002 that Michael Grieves presented the initial conceptual model of DTs during his presentation on product life cycle management for manufacturing. Since then, the use of DTs has expanded to various fields of application (multiphysical, multiscale, and probabilistic simulations) [13].

In 2014, Michael Grieves provided a general definition of the DT concept and identified three major elements of DTs [14-16]: (1) a real space representing a physical object (an object, a process, a person, or a phenomenon), (2) a virtual space containing a virtual object, and (3) a digital thread connecting the real space and the virtual space.

The primary idea is to model these systems computationally to develop and test them more quickly and economically and with fewer potential negative consequences than in real life. DT technology was the third most trending technology in 2020 according to the Institute of Electrical and Electronics Engineers Computer Society [17], where technology experts reveal their annual forecasts for the most widely adopted future technological trends.

However, the current use of DTs remains well below their potential, particularly in the field of life sciences. The development and creation of DTs require a broad understanding of each part of a system, the relationships between these parts, and the analytical power to assess the effect of variables introduced into the system. Currently, there is only a trend toward using DTs in health care. DTs hold great potential, especially in precision medicine, where they can be used to simulate individual therapies and visualize potential therapeutic outcomes and disease progression for each patient [18].

## DTs in Health Care

In health care, a DT is a virtual copy of a physical object or process, such as a patient, their anatomical structure, or a hospital environment. Currently, DTs in health care can leverage numerous data sources, including electronic health records, disease registries, and omics data, as well as physical markers, demographic data, and a person’s lifestyle data over time [19,20].

### DTs and Personalized Health Care

A key feature of a DT is its ability to predict how the object or process will function, with predictions gradually increasing in accuracy through integration with other technologies such as AI. This leads to forecasted results being shared as part of feedback to the original physical entity. Having a dynamic bidirectional flow of information between physical and virtual products enables this technology to create powerful and complex digital representations of the physical entity, further enhancing in-depth testing and facilitating decision-making without altering or depleting the original physical product. Emerging models suggest that DTs may eventually enable early prediction of disease onset or simulate treatment scenarios in silico; however, these capabilities remain largely theoretical and require further validation [19].

A crucial characteristic of DTs is their dynamic bidirectional mapping. DTs are not a simple 1-way map, a digital shadow, or a simulation model of a real physical entity in the digital domain. Different types of DTs can be designed; for example, one can have twins of the entire human body, a single body system, a single organ, or finer component levels (cellular or molecular). DTs can also be created for a specific disease or disorder or for other relevant organisms (eg, viruses). Composite DTs integrate ≥2 of the aforementioned types, whereas reference twins or proto-twins serve as models or archetypes for constructing more complex and individualized DTs of each type.

The level achieved by a given DT depends on the fidelity of the twin (the level of detail captured in the real world and whether updates to connected instrumentation, integrated learning, intelligence, and autonomy are included).

Close similarities have been observed between data-driven health care practices and modern engineering approaches driven by DTs. The notion of exploiting the virtual model to assess the impact of actions on the physical model resembles what in silico clinical analyses aim to achieve by leveraging the dynamic representation of an individual’s unique details such as molecular state, physiological state, and lifestyle over time in the digital domain [21].

Recent studies have proposed an individualized system for disease care and prevention in Europe through the use of “virtual twins” designed to model European patients’ biology and pathological states using data collected across a wide range of individual-characterizing domains (clinical, imaging, and sensor data) [22]. With computational resources and big data technologies becoming increasingly sophisticated, it has been envisioned that creating such personalized digital models will become easier, allowing health care professionals to test all possible treatments and measures before prescribing them to real patients. Such methods would significantly improve European citizens’ quality of life while reducing costs associated with health care provision.

Human DTs in personalized medicine are constructed to represent organs or microstructures within the body and can be expanded to incorporate external factors, including the surrounding environment and social interactions over an observed period. The use of DTs would help understand various health determinants in the general population.

One of the main research opportunities is to develop a virtual system for disease monitoring and management to better predict, understand, and treat these conditions.

### Research Perspectives in Health: Personalization and Public Health

To identify new epidemiological pathways for improving patient care and surpassing traditional known risk factors, this research project focused on analyzing the experience and perception of diseases by considering all data generated by the health care system and health care professionals, information from specific questionnaires (eg, sociodemographic and lifestyle habits), and omics and environmental data, as well as all possible biological, biochemical, imaging, and tissue data. These data will be coupled with data from the insurance database, which includes a patient’s entire health care pathway.

From biological parameter monitoring to connected objects, we will have a considerable amount of data to better characterize various conditions. The lifelong digital traces that individuals leave—often referred to as the “digitosome”—present a rich resource for precision medicine, offering a granular view of behavioral, biological, and environmental influences on health [23]. The concept of the “digitosome” encompasses the full spectrum of digital traces produced by an individual over the course of their lifetime. This includes data derived from interactions with digital platforms, mobile devices, wearable technologies, environmental sensors, and other connected tools. Collectively, these digital footprints offer a multidimensional view of a person’s behavior, context, and health-related patterns, representing a valuable asset for the advancement of personalized health care and data-driven public health strategies [24]. These data should now be considered a full-fledged “omic” data source akin to genomics, transcriptomics, and metabolomics, allowing for a complete characterization of individuals, diseases, and populations as such data continue to grow over time.

The concept of deep digital phenotyping emerges precisely from the combination of data from a traditional biomedical environment with real-world digital data [25].

Deep digital phenotyping combines granular biomedical profiles with temporally rich, real-world digital signals captured through passive or active sensing from connected devices. This hybrid approach enhances our ability to characterize health states dynamically and inform the development of DTs for personalized care [26,27]. This approach aims to provide a multidimensional and dynamic representation of an individual’s health, incorporating both molecular (eg, genomic and proteomic) and behavioral signals. Deep digital phenotyping supports applications in precision medicine by enabling the development of DTs and predictive models that are informed by both static and temporal data streams [28,29]. It holds particular promise in mental health, chronic disease management, and personalized intervention strategies. The integration of these 2 types of data promises a better and more comprehensive characterization of different forms and presentations of conditions inherent to patients.

The establishment of large cohorts or health data repositories that integrate these various data sources makes this approach feasible. In addition, methods such as multiomic analysis or unsupervised deep learning allow for the simultaneous management and analysis of different data sources of diverse natures and structures.

## Data Sources for DTs in Clinical Practice

DTs rely on comprehensive datasets that capture biological, behavioral, and environmental factors affecting health outcomes.

**Figure 1:**
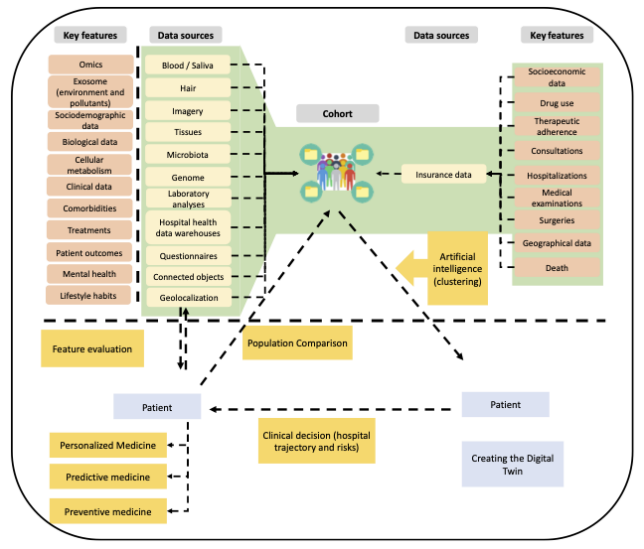
Epidemiological data for the construction of digital twins.

These include (1) omics data (genomics, transcriptomics, proteomics, and metabolomics), (2) exosomes and environmental pollutants (biomarker transporters providing disease insights), (3) sociodemographic data (age, ethnicity, socioeconomic status, and lifestyle factors), (4) biological and clinical data (laboratory test results, imaging, metabolic rates, and physiological parameters), (5) comorbidities and treatment history (chronic diseases, polypharmacy, and therapeutic adherence), (6) health behaviors (physical activity, diet, smoking, and alcohol consumption), (7) tissue-specific data (histopathological analyses and cellular metabolism data), and (8) insurance and geolocation data (access to health care, environmental risks, and spatial epidemiology; Figure 1).

## Mathematical Framework for DTs

### Overview

To construct a DT, we must define mathematical equations modeling individual health trajectories based on multidimensional data inputs.

A DT for clinical practice involves predictive modeling, dynamical systems, and machine learning approaches to simulate and optimize a patient’s health trajectory. The key mathematical formulations needed for constructing a DT using clinical data are presented in this section.

Predictive modeling plays a crucial role in estimating disease risk. A logistic regression model is commonly used to assess the probability of a disease based on patient-specific clinical features such as blood pressure, cholesterol levels, and glucose measurements. These predictive equations leverage regression coefficients derived from population data to enhance the accuracy of risk assessment.

To represent patient health trajectories over time, differential equations are used to capture the evolving state of an individual’s health based on interactions among multiple clinical variables. These models incorporate external influences such as environmental factors, treatment interventions, biological markers, comorbidities, sociodemographic attributes, and genetic predispositions. A simplified linear differential equation can describe the natural progression of health status while integrating the impact of treatments and external influences [30].

For chronic disease modeling, Markov chains are often used to define different health states and transition probabilities [31]. A patient can move between states, such as healthy, predisease, and disease conditions, based on transition matrices, which quantify the likelihood of progression over time. This approach is particularly valuable for conditions such as diabetes and cardiovascular diseases, where disease evolution follows distinct stages.

Bayesian networks facilitate the integration of multiomics data, allowing for the probabilistic modeling of genetic, proteomic, and metabolic interactions [32]. These networks help predict how genetic variations and biological pathways influence disease progression, ultimately guiding personalized treatment decisions. The ability to infer dependencies between biological variables enhances the predictive power of DTs in complex clinical scenarios.

Personalized treatment optimization is achieved through reinforcement learning, which enables AI-driven decision-making in patient care. This method involves selecting optimal treatment actions, such as medication dosages or lifestyle modifications, by maximizing expected patient outcomes over time [33]. Reinforcement learning models continuously refine treatment recommendations based on real-time feedback, ensuring personalized and adaptive health care interventions.

To represent DTs in a structured format, graph neural networks (GNNs) model clinical variables [34,35] as interconnected nodes within a multilayer graph [36]. These networks capture complex dependencies across genomic, clinical, and behavioral data, enabling advanced pattern recognition for precision medicine. The adjacency matrix in a GNN framework encodes relationships between medical parameters, facilitating deep insights into patient health states.

Patient-specific health forecasting is further enhanced through stochastic differential equations, which simulate health trajectories while incorporating uncertainty and variability [37]. These models allow for real-time simulations of different treatment scenarios, providing clinicians with valuable insights into possible health outcomes under various interventions.

Deep learning methodologies, including recurrent neural networks (RNNs) [38,39] and long short-term memory (LSTM) networks [40,41], improve temporal modeling of clinical data by capturing sequential dependencies in patient histories. RNNs process time-series data, whereas LSTM networks mitigate the vanishing gradient problem, enabling accurate long-term health predictions. In addition, transformer-based architectures leverage self-attention mechanisms to prioritize relevant past health states, further refining predictive analytics in DTs.

To quantify patient risk, AI-driven risk score calculations integrate deep learning functions to assess probabilities of adverse health events such as hospitalizations or disease complications. These risk assessments guide proactive interventions and preventive care strategies tailored to individual patients.

Finally, an integrated AI and DT framework combines differential equations with deep learning algorithms to dynamically update patient health trajectories [42]. This hybrid approach enhances predictive accuracy and supports real-time decision-making in clinical settings. As DT technology continues to evolve, its mathematical foundation will drive innovation in personalized medicine, ensuring more precise diagnostics, risk stratification, and treatment optimization.

### Predictive Modeling for Disease Risk

A logistic regression model can be used to estimate the probability *P* of a disease (*y*) based on patient-specific clinical features (*X_i_*):



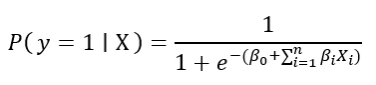



In this equation, *P*(*y* = 1|*X*) is the probability that the outcome *y* equals 1 (disease present) given predictors *X* = [*X*_1_, *X*_2_,..., *X*_n_], *y* is the probability of disease presence (*y*=1) or absence (*y*=0), *X_i_* are patient-specific clinical factors (eg, blood pressure, cholesterol, age, and glucose level), β*_i_* are regression coefficients estimated from population data, and *n* is the total number of predictors included in the model.

### Patient Health Trajectory Over Time (Differential Equation Model)

A dynamical system can be used to describe how a patient’s health status changes over time by modeling the interactions of multiple biological, environmental, and clinical factors. This can be expressed mathematically as follows:



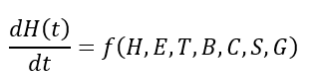



In this equation, 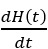 represents the rate of change of a patient’s health status over time (the derivative of *H*(*t*) with respect to time), *H*(*t*) represents the patient’s health status at time *t*, *E* accounts for environmental influences (eg, air pollution and diet), *T* represents treatments or interventions (eg, medications and surgeries), *B* represents biological markers (eg, biomarkers and laboratory test results), *C* represents comorbidities (eg, diabetes and hypertension), *S* denotes sociodemographic factors (eg, income and lifestyle), and *G* accounts for genetic predisposition.

A simplified linear version of the equation can be the following:



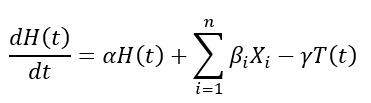



In this equation, 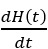 represents the rate of change of a patient’s health status over time (the derivative of *H*(*t*) with respect to time), *H*(*t*) represents the patient’s health status at time *t*, α represents the natural progression of health status, β*_i_* are impact coefficients of clinical variables *X_i_*, and γ represents the effect of treatment *T*(*t*).

### Disease Progression Model (Markov Model)

For chronic conditions such as diabetes or heart failure, disease progression can be modeled using a Markov chain, which captures transitions between discrete health states over time. The basic form of this model is as follows: *P_t_*_+1_ = *P_t_* × *T*, where *P_t_* is a row vector representing the patient’s probability of being in each health state at time *t* (eg, *P_t_*=[0.95, 0.04, 0.01] for a 95% chance of being healthy) and *T* is a transition probability matrix representing the likelihood of disease progression from one state to another.

For example, if there are 3 states (healthy, predisease, and disease conditions), the transition matrix may look like the following:



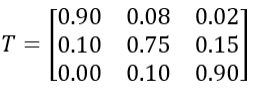



This matrix represents the probabilities of moving from one state to another at each time step.

It can be interpreted as follows: a person who is healthy at time *t* has a 90% chance of remaining healthy, an 8% chance of progressing to the predisease state, and a 2% chance of developing a disease in the next time step. The rows add up to 1, representing the total probability distribution.

### Multiomics Network for Clinical Decision Support (Bayesian Network)

Gene-gene, protein-protein, or metabolite interactions, often derived from multiomics datasets (eg, genomics, transcriptomics, and proteomics), can be represented using a Bayesian network. These models capture conditional dependencies between biological features, enabling personalized inference.

The joint probability distribution of the network is expressed as follows:



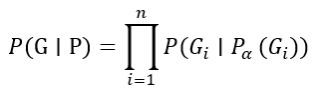



In this equation, *G_i_* represents a gene, protein, or metabolite; *P*_α_(*G_i_*) represents its parent nodes (genes that influence it); the network learns probabilistic dependencies from clinical data, which are encoded in the conditional probabilities P(Gi∣Pα(Gi))P(Gi∣Pα(Gi)) for each node GiGi; *P*(*G*|*P*) represents the joint probability of observing the multiomics data *G* given previous knowledge *P*; and *n* represents the total number of nodes (biological features) in the network.

This model helps predict how genetic variations influence disease progression or treatment response.

### Personalized Treatment Optimization (Reinforcement Learning Approach)

DTs can optimize personalized treatment strategies using reinforcement learning, an AI technique that selects actions to maximize expected health outcomes over time. This is formalized using the Bellman equation:



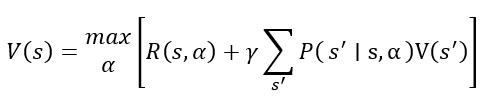



In this equation, *V*(*s*) represents the expected patient health outcome in state *s*, α represents treatment actions (eg, medication dosage and lifestyle changes), *R*(*s*, α) is the immediate reward (eg, improvement in patient health) when action α is taken in state *s*, *P*(*s'*|*s*, α) is the transition probability from state *s* to *s'* given action α, γ is the discount factor that prioritizes long-term health benefits, and γ∈[0,1] represents the discount factor balancing short-term versus long-term health benefits (a higher γ values long-term outcomes, eg, avoiding complications).

This model enables AI-driven treatment recommendations by selecting interventions that maximize patient outcomes.

### Multilayer DT Representation (GNN Model)

A DT can be represented as a multilayer graph in which *nodes* represent clinical variables and *edges* represent relationships (eg, comorbidities and functional links) as follows: *H*^(ι+1)^ = σ(*D*^–1^*AH*^(ι)^*W*^(ι)^), where *H*^(ι)^ is the feature matrix at layer ι, where each row encodes patient-specific features; *A* is the adjacency matrix capturing clinical variable dependencies; *D* is the degree matrix encoding how many connections (edges) each node has; *W*^(ι)^ are trainable parameters applied at layer ι; σ represents the activation function (eg, rectified linear unit), introducing nonlinearity; and *H*^(ι+1)^ represents the updated node features passed to the next layer.

This model allows for complex pattern recognition across clinical, genomic, and behavioral data.

### DT Simulation for Patient-Specific Forecasting

A stochastic differential equation can simulate a patient’s health trajectory with uncertainty as follows: *dH*(*t*) = μ(*H*, *X*, *T*)*dt* + σ(*H*, *X*, *T*)*dW_t_*, where *H*(*t*) represents the patient’s health status at time *t*; μ(*H*, *X*, *T*) is the drift term (expected health trend over time), expected average trajectory, or trend of health over time based on covariates *X* and treatment input *T*; σ(*H*, *X*, *T*) is the diffusion term capturing variability, uncertainty, or randomness in how health changes over time; *dW_t_* is a Wiener process (random fluctuation, also known as Brownian motion) that models random fluctuations (eg, unexpected complications or environmental noise); *X* represents patient-specific characteristics (eg, comorbidities and biometrics); and *T* represents the treatment or intervention protocol applied at time *t*.

This equation allows for real-time simulation of different treatment scenarios for personalized patient care. To enhance the DT framework, we can integrate an AI model for the prediction of future health events using deep learning, RNNs, and transformer models for forecasting health trajectories.

Many clinical events (eg, disease progression, hospitalizations, and biomarker fluctuations) evolve over time. RNNs are well suited for modeling such temporal sequences.

We can use RNNs to model these sequences as follows: *h_t_* = *f*(*W_h_h_t_*_–1_ + *W_x_X_t_* + *b*), where *h_t_* is the hidden state at time *t* (representing the internal “memory” of the model [patient health context]), *h_t_*_–1_ represents the previous hidden state, *X_t_* is the input vector of clinical features at time *t* (eg, heart rate, laboratory values, and medication dose), *W_h_* and *W_x_* are trainable weight matrices (trainable parameters for temporal and input mappings), *b* is the bias term, and *f* is the nonlinear activation function (eg, hyperbolic tangent function or rectified linear unit).

The output prediction for future events can be calculated as follows: *y_t_* = σ(*W*_0_*h_t_* + *b*_0_), where *y_t_* is the predicted probability of a future health event at time *t*, *W*_0_ and *b*_0_ are trainable parameters, and σ is the sigmoid activation function.

RNNs are useful for short-term predictions, but they suffer from vanishing gradients over long sequences.

### LSTM for Health Forecasting

To address the limitations of standard RNNs in capturing long-term dependencies, LSTM networks introduce internal memory cells and gating mechanisms. These are particularly useful for modeling long-range trends in clinical data (eg, treatment response over weeks or months).

The core equations of an LSTM cell at time *t* are as follows: (1) *f_t_* = σ(*W_f_X_i_* + *U_f_h_t_*_–1_ + *b_f_*; forget gate), (2) *i_t_* = σ(*W_i_X_t_* + *U_i_h_t_*_–1_ + *b_i_*; input gate), (3) *o_t_* = σ(*W_o_X_t_* + *U_o_h_t_*_–1_ + *b_o_*; output gate), (4) *c_t_* = *f_t_* ⊙ *c_t_*_–1_ + *i_t_* ⊙ tanh (*W_c_X_t_* + *U_c_h_t_*_–1_ + *b_c_*; cell state update), and (5) *h_t_* = *o_t_* ⊙ tanh (*c_t_*; hidden state output).

In these equations, *X_t_* represents the input vector at time *t* (eg, vital signs and laboratory values); *h_t_* represents the hidden state (short-term memory); *c_t_* is the cell state that retains long-term information; *W*​ and *U* are trainable weight matrices; *b* represents bias terms; *f_t_*, *i_t_*, and *o_t_* are the forget, input, and output gates, respectively; ⊙ represents the Hadamard (element-wise) product; and tanh represents the hyperbolic tangent activation.

LSTM allows long-term clinical data dependencies to be effectively modeled.

### Transformer-Based Health Event Prediction

For even longer time dependencies, we used transformer models such as the time-series transformer.

### Self-Attention Mechanism



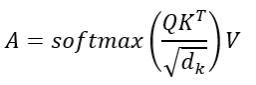



In this equation, *Q* represents the query matrix, which encodes the current time step (eg, current patient state); *K* is the key matrix, which represents all previous time steps (eg, historical health states); *V* represents the value matrix, which carries the information to be aggregated (eg, biometrics and laboratory values); *d_k_* is the scaling factor; *A* is the attention-weighted output, representing a weighted sum of past clinical features; and softmax ensures that the attention weights add up to 1 (ie, probability distribution over time steps).

The transformer learns attention weights to prioritize relevant past health states when predicting future events.

### Prediction Output



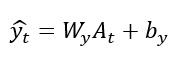



In this equation, 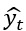 is the probability of a future health event.

### AI-Driven Risk Score Calculation

Using deep learning–based risk scoring, we defined a patient risk function as follows:



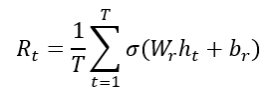



In this equation, *R_t_* is the predicted health risk score (eg, probability of hospitalization); *T* is the number of time steps considered; *h_t_* is the hidden state from a time-series model (eg, RNN, LSTM, or transformer) representing patient health at time *t*; *W_r_* and *b_r_* are learned weights and bias parameters specific to the risk-scoring layer; and σ is the activation function, typically sigmoid, which outputs a probability between 0 and 1.

### Integrated AI+DT Model for Clinical Forecasting

We combined differential equations with deep learning as follows:



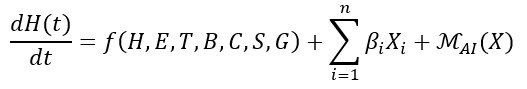



In this equation, *M_AI_*(*X*) is the AI function (RNN, LSTM, or transformer) predicting future health trends.

This function dynamically updates patient health trajectories based on real-time clinical data.

## Methodology of Literature Search for Challenges and Directions of DT

**Figure 2:**
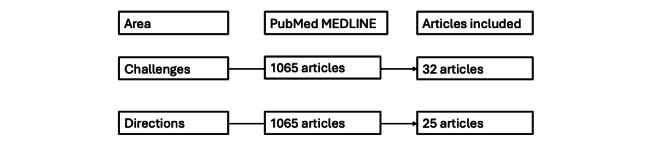
Flowchart of selected articles for the literature review.

A structured review across the major clinical databases (PubMed and MEDLINE; Figure 2) was conducted. The search was performed from inception to March 2025 using Boolean combinations of keywords and Medical Subject Heading terms.

For challenges of DTs, we used the following search string: “digital twin” AND (“benefits” OR “risks” OR “limitations” OR “bias” OR “interpretability” OR “trust”).

For future directions in DTs, we used the following search string: “digital twin” AND (“future perspective” OR “emerging technologies” OR “regulatory framework” OR “personalized medicine” OR “digital health strategy”).

Titles and abstracts were screened for relevance, and a full-text analysis was conducted on a refined subset. Articles were retained if they addressed real-world clinical deployments, systematic analysis of benefits and risks, or conceptual and technological evolutions of DTs in health care. The final corpus was analyzed to extract structured information on implementation type, clinical domain, benefit and risk trade-offs, and forward-looking frameworks.

## Challenges in Implementing DTs in Health Care

Despite the numerous benefits and potential of DTs in clinical management, there are several challenges and limitations that need to be addressed for their successful implementation (Multimedia Appendix 1 [43-74]).

These challenges are outlined in Textbox 1 [43-74].

Addressing these challenges and limitations requires collaborative efforts between health care professionals, researchers, technology developers, regulatory bodies, and policy makers. Investing in infrastructure, data governance, standard development, and validation studies can help overcome these barriers and unlock the full potential of DTs in cardiovascular disease management. Through proactively addressing these challenges, DTs can become powerful tools that enhance risk assessment, disease modeling, treatment optimization, remote patient monitoring, and proactive care, ultimately leading to improved patient outcomes and more personalized cardiovascular health care.

**Challenges to the implementation of digital twins (DTs) in health care.**

**Data privacy and security [43-46]**

The use of DTs involves collecting and analyzing sensitive patient data, raising concerns about privacy and security. Ensuring robust data protection measures, compliance with regulations, and secure data transmission are essential to maintain patient confidentiality and build trust in the technology.

**Data integration and interoperability [47-51]**

DTs rely on the integration of diverse data sources, including electronic health records, wearable devices, and genetic information. However, the interoperability of different data systems and standards remains a challenge, hindering seamless data integration and the full potential of DTs.

**Computational infrastructure and processing power [52-55]**

The processing power and computational infrastructure required to create and run DTs can be substantial. Handling large volumes of data, performing complex calculations, and updating models in real time demand robust infrastructure, which may be a barrier to widespread implementation.

**Validation and accuracy [56-60]**

Ensuring the accuracy and reliability of DT models is crucial for their successful application. Validation studies are necessary to establish the accuracy of predictions, assess the model’s performance against real-world data, and gain confidence in the technology’s effectiveness in clinical practice.

**Ethical and regulatory considerations [61-65]**

As DTs involve making predictions and recommendations based on patient data, ethical considerations such as bias in algorithms, informed consent, and transparency must be addressed. In addition, regulatory frameworks need to adapt to accommodate the unique aspects of DT technology and ensure its responsible use.

**Patient engagement and adoption [66-70]**

The successful implementation of DTs relies on patient engagement and acceptance. Ensuring that patients understand the benefits, risks, and implications of DT technology is essential for their active participation and adoption.

**Cost and accessibility [61,71-74]**

Implementing DTs may require significant financial resources and technical expertise. Ensuring that the technology is accessible and affordable to health care providers across different settings is crucial to ensure equitable access and widespread adoption.

## Future Directions

The future of DTs in clinical management holds great promise, with several exciting directions and advancements on the horizon. The following are some potential future directions for DTs (Multimedia Appendix 2 [43,51,71,74-94]).

### Advanced AI and Machine Learning

Incorporating more advanced AI and machine learning algorithms into DTs can enhance their predictive capabilities. By analyzing large datasets, these algorithms can identify complex patterns; uncover novel insights; and provide more accurate predictions for disease progression, treatment response, and risk assessment [71,75-77].

### Integration of Omics Data

Omics data, including genomics, proteomics, and metabolomics, offer a deeper understanding of the molecular basis of cardiovascular diseases. Integrating these multiomics data into DT models can provide a more comprehensive view of disease mechanisms, facilitate personalized treatment strategies, and enable targeted interventions based on an individual’s unique genetic profile [78,79].

### Real-Time Monitoring and Closed-Loop Systems

Advancements in wearable devices, biosensors, and remote monitoring technologies can enable real-time data collection and integration into DTs. This real-time monitoring can enable closed-loop systems where DTs continuously analyze data and provide feedback to patients and health care providers, facilitating timely interventions and personalized adjustments to treatment plans [74,80-82].

### Virtual Clinical Trials

DTs can play a crucial role in the design and execution of virtual clinical trials. By simulating the effects of interventions on virtual populations, DTs can provide insights into treatment efficacy and safety profiles and identify potential responders or nonresponders. Virtual clinical trials can accelerate the drug development process, reduce costs, and provide more personalized treatment options [83].

### Patient Empowerment and Shared Decision-Making

DTs can empower patients by providing them with personalized insights into their cardiovascular health, treatment options, and lifestyle modifications. Interactive interfaces and decision support tools can facilitate shared decision-making between patients and health care providers, allowing for more patient-centered care and improved treatment adherence [84-86].

### Integration With Health Care Systems and Electronic Health Records

Seamless integration of DTs with existing health care systems and electronic health records is crucial for their widespread adoption. Interoperability standards, data exchange protocols, and integration frameworks need to be developed to ensure the smooth flow of data between DTs and health care providers, enabling the integration of DT technology into routine clinical practice [51].

### Longitudinal Data Analysis and Predictive Modeling

DTs have the potential to leverage long-term longitudinal data to improve predictions and modeling of cardiovascular diseases. By analyzing data trends over time, DTs can provide personalized risk assessments, predict disease progression, and identify optimal treatment strategies for individuals at different stages of the disease [43,87-90].

### Collaborative Research and Data Sharing

Collaboration among researchers, health care providers, and technology developers is essential for advancing the field of DTs in cardiovascular disease management. Data sharing initiatives, research consortia, and interdisciplinary collaborations can accelerate innovation, improve model accuracy, and ensure the ethical and responsible use of DT technology [43,91-94].

## Conclusions

DT technology offers a compelling vision for the future of personalized medicine, yet its potential remains unrealized without a foundation in epidemiological evidence and mathematical modeling. While computational advances have propelled the field, clinical implementation continues to face critical barriers, including fragmented data ecosystems, limited validation, and lack of integration into real-world care.

This viewpoint emphasizes that DTs must move beyond theoretical sophistication and engage directly with the complexities of real populations, longitudinal health trajectories, and clinical variability. Mathematical frameworks such as differential equations, Markov models, and neural networks must be matched with validated epidemiological inputs to ensure robustness, fairness, and clinical utility.

The path forward requires more than innovation, it demands interdisciplinary collaboration; transparent regulatory alignment; and a focus on interpretable, population-representative models. As AI technologies mature and health care systems increasingly rely on data-driven insights, DTs should be seen not only as individualized models but also as public health instruments capable of anticipating risk, informing policy, and enhancing system-wide decision-making.

To achieve this, the community must prioritize standardization, trustworthy data governance, and clinical validation at scale. Only then can DTs transition from promise to practice, reshaping the landscape of modern medicine by making care more predictive, equitable, and responsive to both individuals and populations.

## Appendix

Multimedia Appendix 1 Articles addressing challenges in implementing digital twins in health care.

Multimedia Appendix 2 Articles addressing future directions for digital twins.

## Abbreviations

AI: artificial intelligence
DT: digital twin
GNN: graph neural network
LSTM: long short-term memory
RNN: recurrent neural network
