# Supplementary material for: Digital Twins for Personalized Medicine Require Epidemiological Data and Mathematical Modeling: Viewpoint
Source: J Med Internet Res. 2025 Aug 5;27:e72411. doi: 10.2196/72411 (PMC12365566; doi:10.2196/72411)
Supplement: Multimedia Appendix 1 [file jmir_v27i1e72411_app1.docx]

**Supplementary Table 1.** Articles addressing challenges in implementing digital twins (DTs) in health care.

| Study | Year | Challenge area | Implementation type | Clinical domain | Benefit and risk trade-offs | Main results | Forward-looking frameworks | Summary and relevance |
| --- | --- | --- | --- | --- | --- | --- | --- | --- |
| Boyd et al [43] | 2025 | Data privacy and security | TRE^a^ | Population health data integration | Enables secure access and reuse of data; risk of reidentification | Infrastructure for data federation while preserving privacy | Federated research using TREs | Explores TREs enabling collaborative DT development |
| Fernández-Rhodes and Wagner [44] | 2025 | Data privacy and security | Privacy-respecting data governance | Genomic research | Supports multi-institutional collaboration; complex compliance | Introduces participatory data stewardship model | Collaborative consent framework | Framework for privacy-compliant genetic research with DT implications |
| Giuffrè and Shung [45] | 2023 | Data privacy and security | Synthetic data generator | General health care and machine learning | Enhances data availability for training; may not replicate rare cases accurately | Demonstrates synthetic datasets for secure algorithm development | Use of synthetic data for AI^b^ and DT | Synthetic data as privacy-compliant training input for DTs |
| Ali et al [46] | 2023 | Data privacy and security | Explainable AI in immersive systems | Mixed reality and metaverse in health care | Empowers patient control; risks from decentralized access | Merges DTs with immersive health care; highlights user control | Human-in-the-loop privacy controls | Privacy-sensitive frameworks in immersive AI environments |
| Hong et al [47] | 2024 | Data integration and interoperability | Semantic LLM^c^-based architecture | Cross-domain health and telecommunication systems | Enhances scalability and semantic integration; may increase model complexity | Introduces scalable DT infrastructure based on semantic models | Semantic 5G-enabled twin ecosystems | Integrates semantic model–driven architecture for DT interoperability |
| Ton et al [48] | 2022 | Data integration and interoperability | Semantic segmentation algorithms | Environmental and biomedical data modeling | Improves data harmonization; risk of overfitting or misclassification | Applies deep semantic segmentation for structured data analysis | Semantic learning for DT structuring | Refines semantic classification critical for DT modeling |
| Saba et al [50] | 2023 | Data integration and interoperability | AI-enhanced imaging pipelines | Radiology and imaging | Standardizes multimodal data input; potential lack of cross-vendor compatibility | Outlines AI-driven CT^d^ and MR^e^ harmonization techniques | Imaging pipeline standardization | Provides protocols for data input harmonization in radiological DTs |
| Vogel-Heuser et al [49] | 2021 | Data integration and interoperability | Semantic-analytical integration | Biomedical engineering | Enables deep integration of heterogeneous data; may require cross-discipline coordination | Fuses semantic web technology and analytics for digital engineering twins | Semantic-analytics twin integration | Combines data mapping and semantic reasoning across engineering-health boundaries |
| Sun et al [51] | 2020 | Data integration and interoperability | Standardized industry architecture | Industrial-health convergence | Boosts interoperability and system integration; requires broad compliance | Proposes interoperability framework across DT platforms | Standardization protocols | Architecture design for harmonizing DT ecosystems in industry and health |
| Materne et al [52] | 2025 | Computational infrastructure | Microvascular simulation DT | Neurovascular health | Detailed physiology modeling; burden of high-resolution data processing | Modeled brain microvasculature using patient-specific simulations | Microscale DTs in precision neurocare | Personalized high-resolution brain twin modeling |
| Gillgallon et al [53] | 2025 | Computational infrastructure | Distributed DT simulator | Health and 6G simulation | Scalable to complex networks; requires advanced orchestration | Introduced SimulatorOrchestrator platform for managing large-scale DTs | 6G-ready DT orchestration | Cloud-based scalable DT platform |
| Sauro et al [54] | 2025 | Computational infrastructure | Computational guidelines (FAIR^f^ to CURE^g^) | Biomedical modeling | Improves reproducibility; demands strict modeling compliance | Outlined standards for robust and sustainable DT modeling | From FAIR to CURE protocols | Promotes robust DT development through reproducibility frameworks |
| Zappon et al [55] | 2025 | Computational infrastructure | End-to-end cardiac simulation | Cardiology | Supports real-time modeling; requires tailored processing frameworks | Enabled near–real-time cardiac DT simulations | Real-time DT optimization pipelines | Full simulation pipeline for heart twins |
| Kimpton et al [56] | 2025 | Validation and accuracy | Uncertainty quantification protocol | Model reliability | Increases transparency and trust; adds modeling complexity | Highlights UQ^h^ methods to improve DT confidence | Standardized UQ in DT validation | Highlights key techniques for increasing confidence in DT model outputs through uncertainty analysis |
| Wang et al [57] | 2025 | Validation and accuracy | Predictive modeling improvement | Renewable energy forecasting | Boosts model precision; applicability to health requires adaptation | Enhanced forecasting accuracy with potential DT transfer | Optimized prediction algorithms | Demonstrates enhanced accuracy in predictive modeling with relevance to improving DT performance |
| Sprint et al [58] | 2024 | Validation and accuracy | Longitudinal sensing DT | General health monitoring | Real-time tracking enables better personalization; may lead to data overload | Integrated DT with longitudinal biometric sensing | Continuous sensing for DTs | Focuses on validating DTs via continuous real-time sensing data in health care contexts |
| Klonoff et al [59] | 2024 | Validation and accuracy | Clinical accuracy grid | Diabetes monitoring | Framework for accurate data interpretation; may need disease-specific calibration | Proposed validated grid for noninvasive monitors | Standardized DT feedback models | Introduces a clinically validated accuracy grid framework applicable to DT monitoring systems |
| Antipas et al [60] | 2024 | Validation and accuracy | Pharmaceutical simulation framework | Pharmacology | Enables system-agnostic simulations; requires robust assumptions | Validated solubility predictions with DT relevance | Simulation validation pipelines | Explores validation of pharmaceutical modeling feeding into DTs for precision pharmacology |
| Strigari et al [61] | 2025 | Ethical and regulatory considerations | Computational oncology DT | Radiation oncology | High personalization; raises regulatory and ethical scrutiny | Demonstrates advanced DT simulation in oncology | Governance frameworks in simulation | Explores ethical and regulatory issues in DT simulation environments in oncology; emphasizes governance and accountability |
| Yuan et al [62] | 2024 | Ethical and regulatory considerations | Graph neural network integration | Personalized genomic medicine | Boosts personalization; may introduce algorithmic bias | Proposes multi-view graph models for regulatory-sensitive systems | AI transparency in genomics | Discusses regulatory sensitivity of multi-view AI systems underpinning DTs, particularly in personalized medicine |
| Ferlito et al [63] | 2024 | Ethical and regulatory considerations | Ethical policy framework | General digital health | Promotes responsible DT use; challenges in operationalization | Outlines a moral philosophy lens for DT adoption | Bioethical integration road map | Provides a bioethical framework to assess relational and moral responsibilities in DT integration |
| Watson et al [64] | 2024 | Ethical and regulatory considerations | Bias reduction platform | Health care decision-making | Reduces racial bias; depends on data representativeness | Analyzes how DTs and AI alter bias in clinical decisions | Bias-aware DT frameworks | Assesses potential of DTs and AI to mitigate or reproduce racial bias in clinical settings |
| Jabin et al [65] | 2024 | Ethical and regulatory considerations | AI-driven policy audit | Sub-Saharan health systems | Enables context-aware evaluation; varies by infrastructure maturity | Evaluates ethics and policy gaps in DT uptake in Nigeria | Regulatory harmonization in LMICs^i^ | Reviews ethical, cultural, and policy considerations in AI and DT technologies across health systems |
| Budin-Ljøsne et al [70] | 2024 | Patient engagement and adoption | Longitudinal participation model | Digital health research | Enhances patient trust and engagement; requires sustained interaction | Outlines models for participant-driven digital health engagement | Participatory DT development | Outlines strategies to improve patient involvement and trust in digital health ecosystems relevant to DT engagement |
| Gonzalez Viejo et al [66] | 2024 | Patient engagement and adoption | User perception survey | Public health technology acceptability | Improves design insights; limited to hypothetical scenarios | Evaluates consumer openness to novel health technology | User-centric DT modeling | Investigates public perception and acceptability of health-linked innovations relevant for understanding DT user engagement |
| Zackoff et al [67] | 2024 | Patient engagement and adoption | Autonomous assistant evaluation | AI interface usability | Increases autonomy and interaction; may challenge user comprehension | Assessed tolerability of virtual assistants in clinical use | Human-machine communication in DTs | Examines acceptability of AI interfaces in health care; parallels DT interaction models regarding user trust and usability |
| Winter and Chico [68] | 2023 | Patient engagement and adoption | NASSS^j^ framework application | General DT integration | Supports multilevel analysis of adoption; implementation dependent | Applied NASSS to identify DT deployment challenges | Sociotechnical DT evaluation | Applies a comprehensive framework to assess barriers to DT adoption, scale-up, and long-term integration |
| Jawad et al [69] | 2022 | Patient engagement and adoption | Adoption analysis model | Manufacturing using DTs | Offers cross-sector insight; limited health care translation | Mapped adoption readiness and stakeholder involvement | Adoption modeling in DT planning | Provides insight into adoption patterns, stakeholder alignment, and readiness for DT deployment across sectors |
| Bearman and Ajjawi [71] | 2025 | Cost and accessibility | AI education framework | Medical education and equity | Promotes equity awareness; requires curricular transformation | Analyzes gender bias in AI training relevant to DT deployment | Inclusive training for DT development | Highlights systemic inequities in AI access and training, with implications for equitable DT deployment |
| Ma et al [72] | 2025 | Cost and accessibility | Infrastructure cost assessment | Environmental health systems | Estimates long-term ROI^k^; limited clinical specificity | Evaluates cost and emission impacts of smart infrastructure | Eco-economic digital infrastructures | Evaluates cost structures in digital infrastructure; relevant to long-term DT system adoption |
| Strigari et al [61] | 2025 | Cost and accessibility | Oncology simulation platform | Oncology | Personalized insights; significant cost for high-fidelity models | Describes resource-intensive DT simulations in cancer treatment | Simulation cost-benefit planning | Explores high-cost simulation models in oncology, providing insights into resource needs for DT scalability |
| van Voorst et al [73] | 2024 | Cost and accessibility | Cost-effectiveness imaging trial | Stroke and neurovascular imaging | Optimizes intervention timing; requires imaging access | Cost-effectiveness model for CT perfusion use in thrombectomy | Precision imaging economics | Provides a health care–specific cost-effectiveness model tied to imaging decisions with parallels to DT implementation |
| Cai et al [74] | 2024 | Cost and accessibility | Real-time surveillance system | Infectious disease monitoring | Reduces outbreak risk; initial deployment cost | Assesses economic viability of real-time alerting | Scalable monitoring via DT logic | Directly addresses cost-effectiveness of digital monitoring frameworks akin to DT alert systems |

^a^TRE: trusted research environment.

^b^AI: artificial intelligence.

^c^LLM: large language model.

^d^CT: computed tomography.

^e^MR: magnetic resonance.

^f^FAIR: Findable, Accessible, Interoperable, Reusable

^g^CURE: Computable, Usable, Reproducible, Extensible

^h^UQ: **Uncertainty Quantification.**

^i^LMIC: low- or middle-income country.

^j^NASS: nonadoption, abandonment, scale-up, spread, and sustainability.

^k^ROI: return on investment.
