# Supplementary material for: Digital Twins for Personalized Medicine Require Epidemiological Data and Mathematical Modeling: Viewpoint
Source: J Med Internet Res. 2025 Aug 5;27:e72411. doi: 10.2196/72411 (PMC12365566; doi:10.2196/72411)
Supplement: Multimedia Appendix 2 [file jmir_v27i1e72411_app2.docx]

**Supplementary Table 2.** Articles addressing future directions for digital twins (DTs).

| Study | Year | Challenge area | Implementation type | Clinical domain | Benefit and risk trade-offs | Main results | Forward-looking frameworks | Summary and relevance |
| --- | --- | --- | --- | --- | --- | --- | --- | --- |
| Li et al [75] | 2025 | Advanced AI^a^ and ML^b^ | Interpretable DL^c^ | Outcome prediction | Increases transparency and explainability; adds complexity | Improves the interpretability of clinical prediction models | Explainable AI frameworks for DTs | Applies interpretable DL models for clinical prediction tasks; highly relevant to explainable DT applications |
| Ocana et al [76] | 2025 | Advanced AI and ML | AI in drug discovery | Pharmacology | Accelerates simulation efficiency; dependent on curated datasets | Improves drug simulation via ML-enhanced pharmacokinetics | ML-powered precision drug twin development | Explores AI integration in pharmacology, forming the backbone of DT-driven simulation in drug development |
| Bearman and Ajjawi [71] | 2025 | Advanced AI and ML | Equity-oriented AI | Medical education | Raises awareness of AI bias; requires system-level reform | Examines bias and equity in AI training | Ethical integration in AI-powered DTs | Examines systemic AI biases and equity concerns with implications for ethical DT integration |
| Zhu et al [77] | 2025 | Advanced AI and ML | Predictive DNNs^d^ | Biomedical filtration | Enables efficient modeling; limited generalizability | Demonstrates membrane fouling prediction using DNNs | Transferable prediction for DTs in health environments | Uses advanced neural networks to model biomedical filtration; applicable to environmental health DTs |
| Ooka [78] | 2025 | Integration of omics data | Genomic ecosystem platform | Preventive medicine | Improves early detection; raises governance concerns | Presents scalable genomic systems for early intervention | Genomics-integrated DT ecosystem | Proposes genomic platforms for scalable integration in preventive DT systems |
| Li et al [79] | 2024 | Integration of omics data | Transcriptomics-based multi-organ modeling | Systems medicine | Enhances biological fidelity; resource intensive | Bridges transcriptomic data to improve multi-organ DT coherence | System-wide omics convergence | Uses organ-level transcriptomics to enable system-wide biological fidelity in DT modeling |
| Shen et al [80] | 2025 | Real-time monitoring and closed-loop systems | Real-time update algorithms | General DT engineering | Maintains model fidelity; risk of model drift | Describes update protocols for maintaining model accuracy | Dynamic DT synchronization | Proposes dynamic updating methods to maintain DT accuracy in real-time scenarios |
| Unoki et al [81] | 2024 | Real-time monitoring and closed-loop systems | Closed-loop ECMO^e^ controller | Critical care | Improves automation in life support; complex safety oversight required | Describes feedback-controlled ECMO for clinical stabilization | Clinical-grade autonomous loops | Automated feedback system for life support; applicable to DT-based critical care systems |
| Le Nepvou De Carfort et al [82] | 2024 | Real-time monitoring and closed-loop systems | Sensor-driven CFD^f^ twin | Bioreactor engineering | Improves efficiency via feedback; model sensitivity to input errors | Uses sensors for closed-loop DT optimization | Feedback-enabled process optimization | Implements sensor-based feedback for real-time process optimization in DT engineering models |
| Cai et al [74] | 2024 | Real-time monitoring and closed-loop systems | Spatiotemporal alert system | Infectious disease control | Improves outbreak detection; initial infrastructure costly | Demonstrates cost-effective disease alert network | Real-time risk surveillance | Applies real-time surveillance to infectious disease management; parallels DT alert architectures |
| Qi and Cao [83] | 2023 | Virtual clinical trials | In silico simulation framework | Pharmacology and drug development | Models’ real-world variability; lacks real-time feedback | Simulates drug efficacy and toxicity using virtual cohorts | Virtual trial pipelines for DT-driven pharmacology | Proposes a computational framework for in silico trials using DTs to model drug responses and patient variability |
| Lin et al [84] | 2024 | Patient empowerment and shared decision-making | Decision support platform | Lung cancer screening | Promotes shared decision-making; patient education needed | Established need for shared decision models in screening programs | DT-assisted clinical communication frameworks | Addresses patient-centered frameworks to facilitate shared decisions in cancer diagnostics; relevant to DT-informed discussions |
| Pinton [85] | 2023 | Patient empowerment and shared decision-making | AI-driven prognostic tools | Prognostic communication | Improves patient autonomy; may challenge traditional authority structures | Analyzed AI’s effect on prognostic clarity and agency | AI-human interaction in prognostic DTs | Explores AI’s influence on prognostic transparency and patient agency, indirectly supporting DT-enhanced autonomy |
| Blasiak et al [86] | 2022 | Patient empowerment and shared decision-making | Omnichannel patient engagement | General patient engagement | Boosts accessibility and responsiveness; digital divide may persist | Implemented virtual assistants in health care platforms | User-centered DT interaction | Demonstrates how integrated digital platforms—including virtual assistants—can improve patient empowerment; relevant to DT interfaces |
| Sun et al [51] | 2020 | Integration with health care systems and EHRs^g^ | Standardized interoperability architecture | Health care systems | Improves integration and scalability; requires broad institutional compliance | Frameworks proposed for DT-EHR data interoperability | Industry 4.0 integration protocols | Proposes frameworks for data interoperability and integration applicable to DT systems and EHRs in health care |
| Giri et al [87] | 2025 | Longitudinal data and predictive modeling | Orthodontic twin model | Pediatric dental development | Improves prediction of orthodontic outcomes; limited by cohort specificity | Modeled developmental variation using longitudinal data | Orthodontic developmental twin prediction | Uses longitudinal data to model developmental variation and predict orthodontic outcomes—applicable to pediatric DTs |
| Boyd et al [43] | 2025 | Longitudinal data and predictive modeling | TRE^h^ | Population health research | Supports data linkage for longitudinal modeling; privacy risks | Enables predictive twin modeling from linked population data | TRE-based longitudinal DT research | Establishes infrastructure for linking longitudinal data to support predictive research, including future DT applications |
| Blemker et al [88] | 2025 | Longitudinal data and predictive modeling | Multi-scale ML model | Aging and musculoskeletal health | Accurate prediction of decline; complex data input requirements | Predicted musculoskeletal decline using multi-scale data | Age-related DT forecasting framework | Presents a predictive ML model based on long-term functional and anatomical data; highly relevant for DT applications in aging |
| Fjell et al [89] | 2025 | Longitudinal data and predictive modeling | Cohort modeling framework | Cognitive aging | Enhances cognitive decline prediction; sensitive to data quality | Used cohort data to project cognitive aging trajectories | Neurocognitive twin modeling | Applies longitudinal cohort modeling to predict trajectories of cognitive aging; potential input for digital brain twins |
| Giri et al [90] | 2024 | Longitudinal data and predictive modeling | Twin study-based framework | Developmental genomics | Explores environmental-genetic interactions; requires long-term tracking | Quantified influence of genetic and environmental factors using twins | Epigenetic-environmental DT modeling | Analyzes longitudinal twin cohort data to quantify influences over time—critical for personalized twin-based simulations |
| Boyd et al [43] | 2025 | Collaborative research and data sharing | TRE | Population health | Enables data sharing with privacy safeguards; requires controlled access | Developed TRE infrastructure for secure longitudinal DT research | Privacy-preserving collaborative DT ecosystem | Presents a TRE framework enabling secure and collaborative longitudinal data sharing |
| Krupas et al [91] | 2024 | Collaborative research and data sharing | Interoperable human-robot DT framework | Human-robot interaction | Supports real-time collaboration; complex system integration | Designed a human-centric DT platform for collaborative robotics | Human-machine integration architecture | Highlights a collaborative architecture using interoperable frameworks for human-machine integration |
| Kostavelis et al [92] | 2024 | Collaborative research and data sharing | Multi-stakeholder robotic platform | Robotics research | Fosters cross-sector collaboration; lacks clinical validation | Showcases collaborative research in DT-guided robotic systems | Multi-actor DT research architecture | Develops a multi-stakeholder platform supporting collaborative research in DT robotics |
| Qu et al [93] | 2023 | Collaborative research and data sharing | DTQFL^i^ | Biomedical AI systems | Protects data ownership and decentralization; increases complexity | Introduced federated DT learning under privacy constraints | Federated quantum DT pipeline | Introduces a federated learning framework combining privacy-preserving AI and collaborative DTs |
| Othman and Yang [94] | 2023 | Collaborative research and data sharing | Human-robot smart manufacturing integration | Industrial smart manufacturing | Facilitates real-time control; requires synchronized standards | Optimized data sharing between human and robotic DT systems | Synchronized robotic DT control | Examines data sharing and synchronization between human and robotic agents in DT contexts |

^a^AI: artificial intelligence.

^b^ML: machine learning.

^c^DL: deep learning.

^d^DNN deep neural network.

^e^ECMO: ExtraCorporeal Membrane Oxygenation

^f^CFD: Computational Fluid Dynamics

^g^EHR: electronic health record.

^h^TRE: trusted research environment.

^i^DTQFL: DT quantum federated learning.
